# Supplementary material for: The impact of community led alternative rite of passage on eradication of female genital mutilation/cutting in Kajiado County, Kenya: A quasi-experimental study
Source: PLoS One. 2021 Apr 28;16(4):e0249662. doi: 10.1371/journal.pone.0249662 (PMC8081212; doi:10.1371/journal.pone.0249662)
Supplement: S1 Data — (DOCX) [file pone.0249662.s001.docx]

# Annexe 1a: In-depth interviews questionnaire – Arp beneficiaries/ recent arp graduates

***Administer this questionnaire to young women, beneficiaries of Amref ARP programme***

| A. | Do you consent to participate in this study? **(Ask after reading the consent form).**  **Yes = 1, No = 2 (stop and thank the interviewee)** | [____] |
| --- | --- | --- |
| B.. | Interviewee number | [____][____] |
| C. | Interviewers Signature |  |
| D. | Date of Interview (DD/MM/YY) | [______/_____/_____] |
| E. | Interviewer Code | [____][____] |

|  | **Respondent’s Background** |  |  |
| --- | --- | --- | --- |
| Q1 | Age | [___]___] |  |
| Q2 | Are you currently enrolled in school?  **Yes = 1 No =2** | [___]___] |  |
| Q3 | What is the highest level of education that you completed?  **No Education = 1 Primary = 2 Secondary = 3 Tertiary =4** | [____] |  |
| Q4 | How many siblings do you have? |  |  |
|  | 1. Sisters | [___]___] |  |
|  | 1. Brothers | [___]___] |  |
| Q5 | 1. What is the gender of the head of the household?   **Male = 1 Female =2** | [___]___] |  |
|  | 1. What is the highest level of education of the head of the household?   **No Education = 1 Primary = 2 Secondary = 3 Tertiary =4** | [____] |  |
| Q6 | Which year did you enrol to the ARP programme? | [__]__][__]__] |  |
| Q7 | Which year did you graduate from the programme? | [__]__][__]__] |  |
| Q8 | Are you married?  **Yes = 1** **No = 2 [skip sequencing]** | [___] |  |
|  | 1. If yes, what was your age at marriage? | [___]___] |  |
|  | 1. Was the decision to get married yours?   **Yes = 1 No = 2** | [___] |  |
|  | 1. If no, who made this decision for you? | |  |
| Q9 | Do you have children? If yes,  **Yes = 1** **No = 2 [skip sequencing]** | [___]___] |  |
|  | 1. How many children do you have? | [___]___] |  |
|  | 1. What was your age at first pregnancy? | [___]___] |  |
|  | 1. What is the age of your first child? | [___]___] |  |
|  |  |  |  |
| Q10 | 1. What is your general opinion of FGM/C practices in this community? **[probe further for benefits, disadvantages and attitudes, sexual control]** | |  |
|  | 1. What are your views of the effect of FGM/C on the following; 2. Schooling **[probe for completion, retention and performance]** 3. Early/teenage pregnancies 4. Early/child marriage | |  |
| Q11 | How prevalent/widespread is the practice of FGM/C in your community? How many believe it is a good thing? **[probe for age, location, who does it, methods – including medicalisation of process and other camouflaged continuation of the practise]** | |  |
| Q12 | In your opinion   1. Why do some in this community practice (FGM/C) [probe for socio-cultural (transition to womanhood/marriage); and economic benefits (dowry] 2. Why have some abandoned the practise? [probe for socio-cultural (transition to womanhood/marriageability), educational and health reasons] | |  |
| Q13 | Have you seen change regarding FGM/C in your community? [knowledge, attitudes, perceptions, practice] **What and who** is influencing this change? [probe for both ARP and non-ARP influences] | |  |
| Q14 | In your view, should the practice be continued or be stopped? [probe for reasons; who/what influences your reasoning?] | |  |
| Q15 | How did you experience your transition from childhood to womanhood? [probe for how and why] | |  |
| Q16 | What was your highest level of education? In case you did not complete school, what was the reason for drop-out. Did your (non) FGM status have any influence on your schooling? [probe further for the decision to stay in school, performance] | |  |
| Q17 | How did (not) being circumcised influence your views/decision on when to get married? [probe further to establish before and after perceptions] | |  |
| Q18 | How did (not) being circumcised influence your views/decision on when to have children? [probe further] | |  |
| Q19 | What is the attitude of the community towards girls that are not circumcised? | |  |
| Q20 | - 1. What role did you have in making decisions about your life course (e.g. education, marriage, having children, etc)? [probe further for examples of decision making and freedom of choice]   2. What factors inform these decisions? [probe for factors not related to ARP e.g. SRHR] | |  |
| Q21 | In your views, what is the effect of not being circumcised on marriageability compared to one who has undergone FGM/C? [probe further] | |  |
| Q22 | Were you ever in the course of your life thinking of getting circumcised? Why? Is it common to find non-circumcised girls, e.g. ARP graduates revert to FGM/C practices? Why do you think so? | |  |
| Q23 | What is your opinion of the Alternative Rite of Passage programme? [probe further benefits, disadvantages and attitudes] | |  |
| Q24 | What was your personal experience participating in the programme? | |  |
| Q25 | What is your opinion of the ARP programme as an alternative to FGM/C? | |  |
| Q26 | Would you circumcise your daughter? What would you advise girls from your immediate family about circumcision? Why? Would you say the same to other girls from your community? Why? | |  |
|  |  | |  |
| Q27 | Which interventions on FGM influenced your life and why? | | |
| Q28 | Do you have suggestions how these interventions can be improved? Please elaborate [probe for what can be done differently] | | |
| Q29 | Any other comments or information you would like to share? | |  |

Thank you for your time

# Annexe 1b: In-depth interviews questionnaire – non-Arp beneficiaries

***Administer this questionnaire to young women who have not benefited from ARP***

| A. | Do you consent to participate in this study? (After reading the consent form).  **Yes = 1, No = 2 [stop and thank the interviewee]** | [____] |
| --- | --- | --- |
| B. | Interviewee number | [____][____] |
| C. | Interviewers Signature |  |
| D. | Date of Interview (DD/MM/YY) | [______/_____/_____] |
| E. | Interviewer Code | [____][____][____] |

|  | **Respondent’s Background** |  |
| --- | --- | --- |
| Q1 | Age | [____] |
| Q2 | Are you currently enrolled in school?  **Yes = 1 No =2** | [___]___] |
| Q3 | What is the highest level of education that you completed?  **No Education = 1 Primary = 2 Secondary = 3 Tertiary =4** | [____] |
| Q4 | How many siblings do you have? |  |
|  | 1. Sisters | [___]___] |
|  | 1. Brothers | [___]___] |
| Q5 | 1. What is the gender of the head of the household?   **Male = 1 Female =2** | [___] |
|  | 1. What is the highest level of education of the head of the household?   **No Education = 1 Primary = 2 Secondary = 3 Tertiary = 4** | [____] |
| Q6 | Are you married?  **Yes = 1 No = 2 [skip sequencing]** | [___]___] |
|  | 1. If yes, what was your age at marriage? | [___]___] |
|  | 1. Was the decision to get married yours?   **Yes = 1 No = 2** | [___]___] |
|  | 1. If no, who made this decision for you? | |
| Q7 | Do you have children? If yes  **Yes = 1 No = 2 [skip sequencing]** | [___]___] |
|  | 1. How many children do you have? | [___]___] |
|  | 1. What was your age at first pregnancy? | [___]___] |
|  | 1. What is the age of your first child? | [___]___] |
|  |  |  |
| Q8 | 1. What is your general opinion of FGM/C practices in this community? **[probe further for benefits, disadvantages and attitudes, beliefs e.g sexual control]** | |
|  | 1. What are your views of the effect of FGM/C on the following; 2. Schooling **[probe for completion, retention and performance]** 3. Early/teenage pregnancies 4. Early, Child and Forced Marriages | |
| Q9 | How prevalent/widespread is the practice of FGM/C in your community? **[probe for age, location, who does it, methods – including medicalisation of process and other camouflaged continuation of the practise]** | |
| Q10 | In your opinion;   1. Why do some in this community practice (FGM/C) **[probe for socio-cultural -marriage; and economic benefits -dowry]** 2. Why have others abandoned the practise? [probe for socio-cultural, educational and health reasons] | |
| Q11 | 1. In your view, what is the attitude of the community towards girls that have been circumcised compared to those that haven’t? | |
|  | 1. What is your opinion of girls in your community that are not circumcised? e.g. when you see the girls in the ARP celebrations: what are your thoughts? | |
| Q12 | In your view, should the practice be continued or be stopped? **[probe for reasons]** | |
| Q13 | What is your opinion of the Alternative Rite of Passage programme compared to FGM/C? **[probe further benefits, disadvantages and attitudes]** | |
| Q14 | What role did you have in deciding on your rite of passage? **[probe for factors that influenced this decision]** | |
| Q15 | How did your rite of passage influence your schooling? **[probe further for the decision to stay in school, performance]** | |
| Q16 | How did your rite of passage influence your views/decision on when to get married? **[probe further to establish before and after perceptions]** | |
| Q17 | How did your rite of passage influence your views/decision on when to have children? **[probe further]** | |
| Q18 | In your view, what is the effect of ARP on marriageability of a graduate compared to one who has undergone FGM/C? **[probe further]** | |
| Q19 | Is it common to find ARP graduates revert to FGM/C practices? Why do you think so? | |
| Q20 | What would you tell girls from your immediate family about ARP programme? Why? Would you say the same to other girls from your community? Why? | |
| Q21 | Any other comments or information you would like to share? | |

Thank you for your time

# Annexe 2a: Key informant interview questionnaire chief

| A. | Do you consent to participate in this study? **[Ask, after reading the consent form**].  **Yes = 1, No = 2 [stop and thank the interviewee]** | [____] |
| --- | --- | --- |
| B. | Interviewee number | [____][____] |
| C. | Position |  |
| D. | Interviewers Signature |  |
| E. | Date of Interview (DD/MM/YY) | [______/_____/_____] |
| F. | Interviewer Code | [____][____][____] |

|  |  |
| --- | --- |
| Q1 | What is your view of FGM/C practice in this community? How widespread is the practice? |
| Q2 | How prevalent/widespread is the practice of FGM/C in this community? **[probe for age, location, who does it, methods – including medicalisation of process and other camouflaged continuation of the practise]** |
| Q3 | What in your assessment is the level of awareness among community members on laws prohibiting FGM/C practices? (Probe if the community understand why some want FGM/C to be eradicated) |
| Q4 | How have you been involved in creating awareness or enforcing laws prohibiting the practise? |
| Q5 | What is your stand towards FGM/C? Do you support or are against the practice? Do you speak up about this? Why (not)? What has been the attitude of the community towards your stand? **[probe further to assess opinions on various groups based on age/sex and any other]** |
| Q6 | What are some of the FGM/C related conflicts that you have to deal with in your line of duty? How do you support girls at the centre of these conflicts, both circumcised and uncircumcised? |
| Q7 | 1. What is your opinion of ARP as an alternative to FGM/C? **[probe on whether it addresses the community’s needs on the rite of passage and its advantages/disadvantages]** 2. Which ARP interventions are you aware of that have been implemented in this community? (Probe for interventions other than AMREF) |
| Q8 | 1. Do you think the FGM prevalence in your community has changed over the past years? How, why and what factors do you think have influenced this change? 2. What impact, if any, has the change in FGM prevalence had on individual girls, families and the community at large? Can you give examples? **[probe on the how and ask for examples]** |
| Q9 | Do you see any difference in the education level among circumcised vs uncircumcised girls: how come? How has not being circumcised/reduction of FGM/C affected the schooling of girls in the community? Why? **[probe for completion, retention, performance]** |
| Q10 | Do you see any difference in the teenage pregnancy rate among circumcised vs uncircumcised girls: how come? How has reduction of FGM/ affected early and teenage pregnancies in the community? Why? **[probe further how and why]** |
| Q11 | Do you see any difference in early and forced among circumcised vs uncircumcised girls: how come? How has reduction of FGM/ affected the early marriage of girls in the community? Why? **[probe further for how and why]** |
| Q12 | Current beliefs and attitudes are informing FGM/C practices. What, in your view, has been the role of interventions to end FGM/C in shaping these beliefs and attitudes? **[enumerate these beliefs/attitudes]** |
| Q13 | Is it common to find uncircumcised girls revert to FGM/C practices? Why do you think so? |
| Q14 | Do you have suggestions how FGM/C can better be eradicated? Please elaborate How can interventions improve? What is already going well? |
| Q15 | What would you say are the lessons learnt in efforts to eradicate FGM/C practice in this community? |
| Q16 | Any other comments or information you would like to share? |

Thank you for your time

# Annexe 2b: Key informant interview questionnaire – member of county assembly

| A. | | Do you consent to participate in this study? **[Ask, after reading the consent form**].  **Yes = 1, No = 2 [stop and thank the interviewee]** | [____] |  |
| --- | --- | --- | --- | --- |
| B. | | Interviewee number | [____][____] |  |
| C. | | Position |  |  |
| D. | | Interviewers Signature |  |  |
| E. | | Date of Interview (DD/MM/YY) | [______/_____/_____] |  |
| F. | | Interviewer Code | [____][____][____] |  |
|  |  | | | |
| Q1 | What is your view of FGM/C practice in this community? How widespread is the practice? | | | |
| Q2 | What efforts if any are there in creating awareness among community members on laws prohibiting FGM/C practices? (Probe if the community understand why some want FGM/C to be eradicated) | | | |
| Q3 | How involved, legislatively or programme-wise is the county government and and/or your office in addressing FGM/C practise in the community? | | | |
| Q4 | What is your stand towards FGM/C? Do you support or are against the practice? Do you speak up about this? Why (not)? What has been the attitude of the community towards your stand? **[probe further to assess opinions on various groups based on age/sex and any other]** | | | |
| Q5 | 1. What is your opinion of ARP as an alternative to FGM/C? **[probe on whether it addresses the community’s needs on the rite of passage and its advantages/disadvantages]** 2. Which ARP interventions are you aware of that have been implemented in this community? (Probe for interventions other than AMREF) | | | |
| Q6 | What in your view is the attitude of the community towards girls who have gone through the ARP programme? How have you been involved in supporting these girls? | | | |
| Q7 | 1. Do you think the FGM prevalence in your community has changed over the past years? How, why and what factors do you think have influenced this change? 2. What impact, if any, has the change in FGM prevalence had on individual girls, families and the community at large? Can you give examples? **[probe on the how and ask for examples]** | | | |
| Q8 | Do you see any difference in the education level among circumcised vs uncircumcised girls: how come? How has not being circumcised/reduction of FGM/C affected the schooling of girls in the community? Why? **[probe for completion, retention, performance]** | | | |
| Q9 | Do you see any difference in the teenage pregnancy rate among circumcised vs uncircumcised girls: how come? How has reduction of FGM/ affected early and teenage pregnancies in the community? Why? **[probe further how and why]** | | | |
| Q10 | Do you see any difference in early and forced among circumcised vs uncircumcised girls: how come? How has reduction of FGM/ affected the early marriage of girls in the community? Why? **[probe further for how and why]** | | | |
| Q11 | Current beliefs and attitudes are informing FGM/C practices. What, in your view, has been the role of interventions to end FGM/C in shaping these beliefs and attitudes? **[enumerate these beliefs/attitudes]** | | | |
| Q12 | Do you have suggestions how FGM can better be eradicated? Please elaborate How can interventions improve? What is already going well? | | | |
| Q13 | What would you say are the lessons learnt in efforts to eradicate FGM/C practice in this community? | | | |
| Q14 | Any other comments or information you would like to share? | | | |

# Annexe 2c: Key Informant interviews – Child protection officer

| A. | Do you consent to participate in this study? **[Ask, after reading the consent form**].  **Yes = 1, No = 2 [stop and thank the interviewee]** | [____] |
| --- | --- | --- |
| B. | Interviewee number | [____][____] |
| C. | Interviewers Signature |  |
| D. | Date of Interview (DD/MM/YY) | [______/_____/_____] |
| E. | Interviewer Code | [____][____][____] |

|  |  |  |
| --- | --- | --- |
| Q1 | What is your opinion of FGM/C practices in this community? **[probe for prevalence, benefits/disadvantages (social, economic, cultural – household decisions), usefulness for girls undergoing the practice]** | |
| Q2 | How prevalent/widespread is the practice of FGM/C in your community? **[probe for age, location, who does it, methods – including medicalisation of process and other camouflaged continuation of the practise]** | |
| Q3 | What are your opinions of programmes/policies/projects to end FGM/C in your community in as far as they seek to protect the rights and well-being of girls in the community? **[Note to enumerator: give examples of these programmes/policies/projects]** | |
| Q4 | Research in Kajiado suggests that young girls are likely to go through FGM/C if their parents have. What are your thoughts about this? What are the experiences on the ground? | |
| Q5 | Some parents threaten to disown their daughters should they not go through FGM/C. Why do you think this is so and what do you think can be done about it? What support do you give to such girls? | |
| Q6 | What are some of the FGM/C related conflicts that you have to deal with in your line of duty? How do you support girls at the centre of these conflicts, both circumcised and uncircumcised? | |
| Q7 | What is the attitude of the community towards girls that have participated in FGM/C compared to those that have not? | |
| Q8 | Do you know girls in your community who are uncircumcised? What have you observed about these girls? Can you give examples of positive/negative factors that these girls face in their lives? How is it different from circumcised girls? **[probe for what and how]** | |
| Q9 | What is your opinion of ARP programme as an alternative to FGM/C? **[probe on whether it addresses the community’s needs on the rite of passage and its advantages/disadvantages]** | |
| Q10 | What has been the attitude of immediate and extended family members on girls that have participated in the ARP programme? **[probe for experience on enrolment, during and post-graduation]** | |
| Q11 | Do you see any difference in the education level among circumcised vs uncircumcised girls: how come? How has not being circumcised/reduction of FGM/C affected the schooling of girls in the community? Why? **[probe for completion, retention, performance]** | |
| Q12 | Do you see any difference in the teenage pregnancy rate among circumcised vs uncircumcised girls: how come? How has reduction of FGM/ affected early and teenage pregnancies in the community? Why? **[probe further how and why]** | |
| Q13 | Do you see any difference in early and forced among circumcised vs uncircumcised girls: how come? How has reduction of FGM/ affected the early marriage of girls in the community? Why? **[probe further for how and why]** | |
| Q14 | Is it common in this community to find ARP graduates revert to FGM/C practices? Why do you think this is so? | |
| Q15 | Current beliefs and attitudes are informing FGM/C practices. What, in your view, has been the role of interventions to end FGM/C in shaping these beliefs and attitudes? **[enumerate these beliefs/attitudes]** | |
| Q16 | What would you say has worked or not worked well in this programme and why? **[probe for reasons and suggestions for improvements]** | |
| Q17 | What would you say are the lessons learnt in efforts to eradicate FGM/C practice in this community? | |
| Q18 | Can you give a story about a girl (between 10-25 year) in your community, circumcised or not, that is remarkable to you? | |
| Q19 | Any other comments or information you would like to share? | |

Thank you for your time

# Annexe 2d: Key Informant Interviews – Head teachers

| A. | Do you consent to participate in this study? **[Ask, after reading the consent form**].  **Yes = 1, No = 2 [stop and thank the interviewee]** | [____] |
| --- | --- | --- |
| B. | Interviewee number | [____][____] |
| C. | Interviewers Signature |  |
| D. | Date of Interview (DD/MM/YY) | [______/_____/_____] |
| E. | Interviewer Code | [____][____][____] |

|  |  |  |
| --- | --- | --- |
| Q1 | What is your opinion of FGM/C practices in this community? **[probe for prevalence, benefits/disadvantages (social, economic, cultural – household decisions), usefulness for girls undergoing the practice]** | |
| Q2 | What is your opinion of programmes/policies/projects to end FGM/C in this community? **[Note to enumerator: give examples of these programmes/policies/projects]** | |
| Q3 | What is your opinion on the ARP programme as an alternative to FGM/C? **[probe on whether it addresses the community’s needs on the rite of passage and its advantages/disadvantages]** | |
| Q4 | Some parents threaten to disown their daughters should they not go through FGM/C. Why do you think this is so and what do you think can done about it? How do you support such girls? | |
| Q5 | How do community attitudes and perceptions towards circumcised and circumcised girls shape general attitudes towards education in this community? | |
| Q6 | 1. How do you/your school support both circumcised and uncircimsized girls in their schooling? 2. What has been the attitude of the community towards these efforts? | |
| Q7 | Do you see any difference in the education level among circumcised vs uncircumcised girls: how come? How has not being circumcised/reduction of FGM/C affected the schooling of girls in the community? Why? **[probe for completion, retention, performance]** | |
| Q8 | Do you see any difference in the teenage pregnancy rate among circumcised vs uncircumcised girls: how come? How has reduction of FGM/ affected early and teenage pregnancies in the community? Why? **[probe further how and why]** | |
| Q9 | Do you see any difference in early and forced among circumcised vs uncircumcised girls: how come? How has reduction of FGM/ affected the early marriage of girls in the community? Why? **[probe further for how and why]** | |
| Q10 | Can you give a story about a girl (between 10-25 year) in your community, circumcised or not, that is remarkable to you? | |
| Q11 | Is it common in this community to find ARP graduates revert to FGM/C practices? Why do you think this is so? | |
| Q12 | What would you say has worked or not worked well in this programme and why? **[probe for reasons and suggestions for improvements]** | |
| Q13 | What would you say are the lessons learnt in efforts to eradicate FGM/C practice in this community? | |
| Q14 | Any other comments or information you would like to share? | |

Thank you for your time

# Annex 2e: Key Informant interviews – cbo/cso official

| A. | Do you consent to participate in this study? **[Ask, after reading the consent form**].  **Yes = 1, No = 2 [stop and thank the interviewee]** | [____] |
| --- | --- | --- |
| B. | Interviewee number | [____][____] |
| C. | Interviewers Signature |  |
| D. | Date of Interview (DD/MM/YY) | [______/_____/_____] |
| E. | Interviewer Code | [____][____][____] |

|  |  |  |
| --- | --- | --- |
| Q1 | What is your opinion of FGM/C practices in this community? **[probe for prevalence, benefits/disadvantages (social, economic, cultural – household decisions), usefulness for girls undergoing the practice]** | |
| Q2 | 1. What is your opinion of programmes/policies/projects to end FGM/C in your community? **[Note to enumerator: give examples of these programmes/policies/projects]** 2. Which of these programmes are you involved in implementing? | |
| Q3 | Research in Kajiado suggests that young girls are likely to go through FGM/C if their parents have. What are your thoughts about this? What has been your experiences on this working with community members? | |
| Q4 | Some parents threaten to disown their daughters should they not go through FGM/C. Why do you think this is so and what do you think can be done about it? What support do you give to such girls? | |
| Q5 | What is the attitude of the community towards girls that have participated in FGM/C compared to those that have not? | |
| Q6 | What is your opinion of ARP programme as an alternative to FGM/C? **[probe on whether it addresses the community’s needs on the rite of passage and its advantages/disadvantages]** | |
| Q7 | Do you know girls in your community who are uncircumcised? What have you observed about these girls? Can you give examples of positive/negative factors that these girls face in their lives? How is it different from circumcised girls? **[probe for what and how]** | |
| Q8 | Do you see any difference in the education level among circumcised vs uncircumcised girls: how come? How has not being circumcised/reduction of FGM/C affected the schooling of girls in the community? Why? **[probe for completion, retention, performance]** | |
| Q9 | Do you see any difference in the teenage pregnancy rate among circumcised vs uncircumcised girls: how come? How has reduction of FGM/ affected early and teenage pregnancies in the community? Why? **[probe further how and why]** | |
| Q10 | Do you see any difference in early and forced among circumcised vs uncircumcised girls: how come? How has reduction of FGM/ affected the early marriage of girls in the community? Why? **[probe further for how and why]** | |
| Q11 | Is it common in this community to find ARP graduates revert to FGM/C practices? Why do you think this is so? | |
| Q12 | Current beliefs and attitudes are informing FGM/C practices. What, in your view, has been the role of interventions to end FGM/C in shaping these beliefs and attitudes? **[enumerate these beliefs/attitudes]** | |
| Q13 | What would you say has worked or not worked well in this programme and why? **[probe for reasons and suggestions for improvements]** | |
| Q14 | What would you say are the lessons learnt in efforts to eradicate FGM/C practice in this community? | |
| Q15 | Can you give a story about a girl (between 10-25 year) in your community, circumcised or not, that is remarkable to you? | |
| Q16 | Any other comments or information you would like to share? | |

# Annexe 2f: Key Informant Interviews – cultural elders

| A. | Do you consent to participate in this study? **[Ask, after reading the consent form**].  **Yes = 1, No = 2 [stop and thank the interviewee]** | [____] |
| --- | --- | --- |
| B. | Interviewee number | [____][____] |
| C. | Interviewers Signature |  |
| D. | Date of Interview (DD/MM/YY) | [______/_____/_____] |
| E. | Interviewer Code | [____][____][____] |

|  |  |  |
| --- | --- | --- |
| Q1 | What is your opinion of FGM/C practice in this community? **[probe for prevalence, benefits/disadvantages (social, economic, cultural – household decisions), usefulness for girls undergoing the practice]** | |
| Q2 | What is your opinion of programmes/policies/projects to end FGM/C in your community? **[Note to enumerator: give examples of these programmes/policies/projects]** | |
| Q3 | What is your stand towards FGM/C? Do you support or are against the practice? Do you speak up about this? Why (not)? What has been the attitude of the community towards your stand? **[probe further to assess opinions on various groups based on age/sex and any other]** | |
| Q4 | 1. Why do some in this community practice (FGM/C) [probe for socio-cultural (transition to womanhood/marriage); and economic benefits (dowry]. 2. Why have some abandoned the practise? [probe for socio-cultural (transition to womanhood/marriageability), educational and health reasons] | |
| Q5 | What is your opinion on medicalisation of the FGM/C? | |
| Q6 | What is your opinion of ARP programme as a replacement to FGM/C? **[probe on whether it addresses the community’s needs on the rite of passage and its advantages/disadvantages]** | |
| Q7 | 1. What has been the effects of ARP programme on the following for girls who have undergone the programme? 2. Schooling **[probe for completion, retention, performance]** Why? 3. Early teenage pregnancies **[probe for prevalence – high/moderate/low]** Why? 4. Child, early and forced marriage **[probe for prevalence – high/moderate/low]** Why? 5. Marriageability **[probe for perceptions and experiences for marriage prospects]** Why? 6. In your view, what other factors other than ARP programme, (if any), have influenced changes observed in (a) above? | |
| Q8 | Do you think the FGM prevalence in your community has changed over the past years? How, why and what factors do you think have influenced this change? | |
| Q9 | What impact, if any, has the change in FGM prevalence had on individual girls, families and the community at large? Can you give examples? **[probe on the how and ask for examples]** | |
| Q10 | Is it common in this community to find ARP graduates revert to FGM/C practices? Why do you think this is so?) | |
| Q11 | Current beliefs and attitudes are informing FGM/C practices. What, in your view, has been the role of interventions to end FGM/C in shaping these beliefs and attitudes? **[enumerate these beliefs/attitudes]** | |
| Q12 | What would you say are the lessons learnt in efforts to eradicate FGM/C practice in this community? | |
| Q13 | Any other comments or information you would like to share? | |

Thank you for your time

# Annexe 2g: Key Informant Interviews – religious leaders

| A. | Do you consent to participate in this study? **[Ask, after reading the consent form**].  **Yes = 1, No = 2 [stop and thank the interviewee]** | [____] |
| --- | --- | --- |
| B. | Interviewee number | [____][____] |
| C. | Interviewers Signature |  |
| D. | Date of Interview (DD/MM/YY) | [______/_____/_____] |
| E. | Interviewer Code | [____][____][____] |

|  |  |  |
| --- | --- | --- |
| Q1 | What is your opinion of FGM/C practice in this community? **[probe for prevalence, benefits/disadvantages (social, economic, cultural – household decisions), usefulness for girls undergoing the practice]** | |
| Q2 | What is your opinion of programmes/policies/projects to end FGM/C in your community? **[Note to enumerator: give examples of these programmes/policies/projects]** | |
| Q3 | What is your stand towards FGM/C? Do you support or are against the practice? Do you speak up about this? Why (not)? What has been the attitude of the community towards your stand? **[probe further to assess opinions on various groups based on age/sex and any other]** | |
| Q4 | 1. Why do some in this community practice (FGM/C) [probe for socio-cultural (transition to womanhood/marriage); and economic benefits (dowry]. 2. Why have some abandoned the practise? [probe for socio-cultural (transition to womanhood/marriageability), educational and health reasons] | |
| Q5 | What is your opinion on medicalisation of FGM/C? | |
| Q6 | What is your opinion of ARP programme as a replacement to FGM/C? **[probe on whether it addresses the community’s needs on the rite of passage and its advantages/disadvantages]** | |
| Q7 | 1. What has been the effects of ARP programme on the following for girls who have undergone the programme? 2. Schooling **[probe for completion, retention, performance]** Why? 3. Early teenage pregnancies **[probe for prevalence – high/moderate/low]** Why? 4. Child, early and forced marriage **[probe for prevalence – high/moderate/low]** Why? 5. Marriageability **[probe for perceptions and experiences for marriage prospects]** Why? 6. In your view, what other factors other than ARP programme, (if any), have influenced changes observed in (a) above? | |
| Q8 | Do you think the FGM prevalence in your community has changed over the past years? How, why and what factors do you think have influenced this change? | |
| Q9 | What impact, if any, has the change in FGM prevalence had on individual girls, families and the community at large? Can you give examples? **[probe on the how and ask for examples]** | |
| Q10 | Is it common in this community to find ARP graduates revert to FGM/C practices? Why do you think this is so?) | |
| Q11 | Current beliefs and attitudes are informing FGM/C practices. What, in your view, has been the role of interventions to end FGM/C in shaping these beliefs and attitudes? **[enumerate these beliefs/attitudes]** | |
| Q12 | What would you say are the lessons learnt in efforts to eradicate FGM/C practice in this community? | |
| Q13 | Any other comments or information you would like to share? | |

# Annexe 2h: Key Informant Interviews – tba (cutters)

**Instructions:**

| A. | | Do you consent to participate in this study? **[Ask, after reading the consent form**].  **Yes = 1, No = 2 [stop and thank the interviewee]** | [____] | |  |
| --- | --- | --- | --- | --- | --- |
| B. | | Interviewee number | [____][____] | |  |
| C. | | Interviewers Signature |  | |  |
| D. | | Date of Interview (DD/MM/YY) | [______/_____/_____] | |  |
| E. | | Interviewer Code | [____][____][____] | |  |
|  | |  | | |  |
| Q1 | | What is your opinion of FGM/C practices in this community? Do you believe the cut is a good/bad thing? Do you cut yourself? **[probe for prevalence, benefits/disadvantages (social, economic, cultural – household decisions), usefulness for girls undergoing the practice]** | | | |
| Q2 | | 1. What is your opinion of programmes/policies/projects to end FGM/C in your community? **[Note to enumerator: give examples of these programmes/policies/projects]** 2. How have these policies/programmes/projects affected your work? **[probe for positive or negative effect]** | | | |
| Q3 | | Some parents threaten to disown their daughters should they not go through FGM/C. Why do you think this is so and what can be done about it? | | | |
| Q4 | | What is the attitude of the community towards girls that have participated in FGM/C? | | | |
| Q5 | | What is your opinion of ARP programme as a replacement to FGM/C? **[probe on whether it addresses the community’s needs on the rite of passage and its advantages/disadvantages]** | | | |
| Q6 | | What is the attitude of the community towards girls that have participated in the ARP programme? | | | |
| Q7 | | What has been the effects of ARP programme on;   1. Your work **[probe for changes in demand]** Why? 2. Your standing in the family and community **[probe for attitudes/acceptance]** Why?   Livelihood **[probe for effect on income generating activities]** Why? | | | |
| Q8 | | Is it common in this community to find ARP graduates revert to FGM/C practices? Why do you think this is so? | | | |
| Q9 | | What would you say has worked or not worked well in this programme and why? **[probe for reasons and suggestions for improvements]** | | | |

Thank you for your time

# Annexe 3a: Focus group discussions questionnaire – adolescent girls & young women

**Instructions:**

1. Give a culturally appropriate welcome to group members.
2. Introduce yourself and any other accompanying member of the study group.
3. Allow participants to introduce themselves
4. Make sure participants introduce themselves before they speak
5. Provide context of the study and explain why it is crucial.
6. Read out the consent form to the group and explain confidentiality.
7. Seek participants consent to participate in the study (read the consent form to the group)
8. Control the discussion to allow equal participation and enable participants to speak freely
9. Do not interrupt when participants speak

**Research Assistant name:**

**Supervisor’s name:**

**Start time:**

**End time:**

|  | **Participant Code** | **Age** | **Gender** |
| --- | --- | --- | --- |
| 1 |  |  |  |
| 2 |  |  |  |
| 3 |  |  |  |
| 4 |  |  |  |
| 5 |  |  |  |
| 6 |  |  |  |
| 7 |  |  |  |
| 8 |  |  |  |
| 9 |  |  |  |
| 10 |  |  |  |
| 11 |  |  |  |
| 12 |  |  |  |

|  |  |  |
| --- | --- | --- |
| Q1 | How is FGM/C viewed in this community**?** **[probe for prevalence, benefits/disadvantages (social, economic, cultural – household decisions), usefulness for girls undergoing the practice]** | |
| Q2 | Some people believe that girls who have not undergone FGM/C are not well prepared for marriage. **[probe for marriageability, chastity, risk of infection, readiness for childbearing]** | |
| Q3 | What opinions/views exist among community members on other approaches/forms/processes of undertaking FGM/C? (**probe for medicalisation/clinical approaches)** | |
| Q4 | What is the opinion of the community about interventions that provide an alternative to FGM/C? **[probe on whether it addresses the community’s needs on the rite of passage and its advantages/disadvantages]** | |
| Q5 | What is the attitude of the community towards girls that are not circumcised? Are uncircumcised girls treated differently from circumcised girls? | |
| Q6 | What are the attitudes of Morans and young men as immediate and extended family members of girls that are uncircumcised? **[probe for experience on enrolment, during and post-graduation]** | |
| Q7 | 1. What has been the effect of not undergoing the cut on the following outcomes for uncircumcised girls? 2. Schooling [probe for completion, retention, performance] Why? 3. Early teenage pregnancies **[probe for prevalence – high/moderate/low]** Why? 4. Child marriage **[probe for prevalence – high/moderate/low]** Why? 5. Marriageability **[probe for perceptions and experiences for marriage prospects]** Why? 6. What other factors other than ARP programme explain the effect observed in (a) above? | |
| Q8 | Is it common in this community to find uncircumcised girls revert to FGM/C practices? | |
| Q9 | What would you say has worked or not worked well in eradicating FGM/C in the community and why? **[probe for reasons and suggestions for improvements]** | |

Thank you for your time

# Annexe 3b: Focus group discussions questionnaire – Morans and young men

**Instructions:**

1. Give a culturally appropriate welcome to group members.
2. Introduce yourself and any other accompanying member of the study group.
3. Allow participants to introduce themselves
4. Make sure participants introduce themselves before they speak
5. Provide context of the study and explain why it is crucial.
6. Read out the consent form to the group and explain confidentiality.
7. Seek participants consent to participate in the study (read the consent form to the group)
8. Control the discussion to allow equal participation and enable participants to speak freely
9. Do not interrupt when participants speak

**Research Assistant name:**

**Supervisor’s name:**

**Start time:**

**End time:**

|  | **Participant Code** | **Age** | **Gender** |
| --- | --- | --- | --- |
| 1 |  |  |  |
| 2 |  |  |  |
| 3 |  |  |  |
| 4 |  |  |  |
| 5 |  |  |  |
| 6 |  |  |  |
| 7 |  |  |  |
| 8 |  |  |  |
| 9 |  |  |  |
| 10 |  |  |  |
| 11 |  |  |  |
| 12 |  |  |  |

|  |  |  |
| --- | --- | --- |
| Q1 | What is the opinion of FGM/C practices in this community? **[probe for prevalence, benefits/disadvantages (social, economic, cultural – household decisions), usefulness for girls undergoing the practice]** | |
| Q2 | What is the opinion on programmes/policies/projects to end FGM/C in your community? **[Note to enumerator: give examples of these programmes/policies/projects]** | |
| Q3 | Some people believe that girls who have not undergone FGM/C are not well prepared for marriage. Do Morans and young men marry uncircumcised girls? why (not)? **[probe for marriageability, chastity, risk of infection, readiness for childbearing]** | |
| Q4 | What opinions/views exist among community members on other approaches/forms/processes of undertaking FGM/C? (**probe for medicalisation/clinical approaches)** | |
| Q5 | What are the opinions of the ARP programme as a replacement to FGM/C? **[probe on whether it addresses the community’s needs on the rite of passage and its advantages/disadvantages]** | |
| Q6 | What is the attitude of the community towards girls that have participated in the ARP programme? | |
| Q7 | What are the attitudes of Morans and young men as immediate and extended family members of girls that have participated in the ARP programme? **[probe for experience on enrolment, during and post-graduation]** | |
| Q8 | Is it common in this community to find ARP graduates revert to FGM/C practices? Why? | |
| Q9 | What has worked or not worked well in this programme and why? **[probe for reasons and suggestions for improvements]** | |

Thank you for your time

# Annexe 3c: Focus group discussions questionnaire – parents

**Instructions:**

1. Give a culturally appropriate welcome to group members.
2. Introduce yourself and any other accompanying member of the study group.
3. Allow participants to introduce themselves
4. Make sure participants introduce themselves before they speak
5. Provide context of the study and explain why it is crucial.
6. Read out the consent form to the group and explain confidentiality.
7. Seek participants consent to participate in the study (read the consent form to the group)
8. Control the discussion to allow equal participation and enable participants to speak freely
9. Do not interrupt when participants speak

**Research Assistant name:**

**Supervisor’s name:**

**Start time:**

**End time:**

|  | **Participant Code** | **Age** | **Gender** | **Child Beneficiary Status (Yes/No)** |
| --- | --- | --- | --- | --- |
| 1 |  |  |  |  |
| 2 |  |  |  |  |
| 3 |  |  |  |  |
| 4 |  |  |  |  |
| 5 |  |  |  |  |
| 6 |  |  |  |  |
| 7 |  |  |  |  |
| 8 |  |  |  |  |
| 9 |  |  |  |  |
| 10 |  |  |  |  |
| 11 |  |  |  |  |
| 12 |  |  |  |  |

|  |  |  |
| --- | --- | --- |
| Q1 | What are the opinions of FGM/C practices in this community? [**probe for prevalence, benefits/disadvantages (social, economic, cultural – household decisions**), usefulness for girls undergoing the practice] | |
| Q2 | What opinions/views exist among community members on other approaches/forms/processes of undertaking FGM/C? (**probe for medicalisation/clinical approaches)** | |
| Q3 | Research in Kajiado suggests that young girls are likely to go through FGM/C if their parents have. Why is this so? | |
| Q4 | How are programmes/policies/projects to end FGM/C viewed in this community? [note to enumerator: give examples of these programmes/policies/projects] | |
| Q5 | Does the community understand why there are efforts including laws that prohibit continuation of FGM/C practice? | |
| Q6 | Some parents threaten to disown their daughters should they not go through FGM/C. Why is this so and what can be done about it? | |
| Q7 | What is the attitude of the community towards girls that have participated in FGM/C? | |
| Q8 | What opinions exists in the community of the ARP programme as an alternative to FGM/C? [probe on whether it addresses the community’s needs on rite of passage and its advantages/disadvantages] | |
| Q9 | What is the attitude of the community towards girls that have participated in the ARP programme? | |
| Q10 | What has been the attitude of immediate and extended family members on girls that have participated in the ARP programme? [probe for experience on enrollment, during and post-graduation] | |
| Q11 | What has been the effects of ARP programme on the following for girls who have undergone the programme?   1. Schooling [probe for completion, retention, performance] Why? 2. Early teenage pregnancies [probe for prevalence – high/moderate/low] Why? 3. Child marriage [probe for prevalence – high/moderate/low] Why? 4. Marriageability [probe for perceptions and experiences for marriage prospects] Why? | |
| Q12 | Is it common in this community to find ARP graduates revert to FGM/C practices? Why do you think this is so?) | |
| Q13 | What would you say has worked or not worked well in this programme and why? [probe for reasons and suggestions for improvements] | |
| Q14 | Any other comments? | |
