# Supplementary material for: The impact of community led alternative rite of passage on eradication of female genital mutilation/cutting in Kajiado County, Kenya: A quasi-experimental study
Source: PLoS One. 2021 Apr 28;16(4):e0249662. doi: 10.1371/journal.pone.0249662 (PMC8081212; doi:10.1371/journal.pone.0249662)
Supplement: Attachment — Submitted filename: PONE-D-20-28794_reviewer (1) - comments.docx [file pone.0249662.s002.docx]

**MANUSCRIPT ID: PONE-D-20-28794: – The Impact of Community Led Alternative Rite of Passage on Eradication of Female Genital Mutilation/Cutting in Kajiado County, Kenya: A quasi-experimental study**

Manuscript is well written and merit worthy. I recommend its consideration for publication

Overall Comment

This manuscript is a quasi-experimental study of community-based interventions on female genital mutilation/cutting (FGM/C) in one region of Kenya among the Maasai who have traditionally conducted the practice universally. The topic of the study is of importance to public health and is also a stated goal of the 2030 Sustainable Development Agenda (goal 5.3). As important as the topic is, it seems there is limited empirical evidence of the effect of FGM/C, or that the evidence is not clear-cut to warrant use of terms like “devastating”. For this, I would advise researchers to consider arguments made in this report [The Public Policy Advisory Network on Female Genital Surgeries in Africa. **Seven Things to Know about Female Genital Surgeries in Africa**. *Hastings Cent Rep*. 2012 Nov-Dec;42(6):19-27.]

Overall, the current study is well done and my assessment is that it is deserving of consideration for publication, which is my recommendation after some considerations by authors. My substantive comments follow.

Abstract:

- Authors should add more details on the Methods statement here. For instance, what type of data is used, where is the data from (e.g., KDHS), what is the sample size, what is the analytical method used (e.g., the DiD)? All that is now missing. The single statement there now on the design of the study is highly uninformative. In general, it is puzzling that the authors seem not to have thought it necessary to include all these other details
- In the Conclusion sub-section more could be stated. For instance, is the CLARP scalable, is it replicable, what is the policy implication?

Background:

- Lines 61-63: can authors add references to this statement
- Lines 76-84: the UNICEF source (and for Figure 1a) that is mentioned here should be cited
- Lines 85-86: cite the UNFPA document here first
- Lines 87-89: the statement says that FGM/C complicates pregnancy and childbirth and “continue to be the leading cause of death among 15- to 19-year-old girls in most parts of the developing world.” There is some contradiction with the statement “in most parts of the developing world” because a previous line (77-79) state the practice is “concentrated across 29 countries”. This is not “most of the developing world”.
- Lines 116-120: does not the KDHS capture types of FGM/C? The quality of references used do not seem strong; later in lines 134-138 data on the different types are noted from the KDHS.
- I think section 1.3 (objectives) and 1.4 (evaluation questions) are repetitive and one can be dropped
- Mention here in this section that ending FGM/C is also part of the Sustainable Development Agenda to 2030 where Goal 5.3 specifically mentions the practice [<https://sdgs.un.org/goals/goal5>] .

Methods:

- The variables “religion” and “ethnicity” are included in the model. Table 2a with KDHS data does not show these, but it is presumed data for these come from the same KDHS; perhaps include these in this Table as well
- Can the authors include sample questionnaires as supplemental files? Since the direct questions are not shown in the results – responses are included – the description of what was contained in these questionnaires under “2.3.2-Data collection tools” is not sufficiently informative
- How have the authors dealt with data from KDHS in years prior to implementation of the County system in 2010? The analysis of quantitative data between treatment county (Kajiado) and control group of counties for all indicators extracted from KDHS would have been collected using regional samples that are different from the county frame. Are the authors using the district frame or other county equivalence? Table 3a seem to show regions by District/sub-county.
- In addition to the KDHS, data from the schools – on educational outcomes/schooling years, completion and retention – in the intervention county (Kajiado) could also be collected and analyzed quantitatively as the records could be available. Perhaps this is still an open research question
- Is it known what the number of beneficiaries of CLARP was? Lines 341-342 states AMREF has a database but the sample was derived “within a school from randomly drawn list of schools” (lines 333-334). The sample size is not shown or stated

Results:

- Please check duplicated text in stating your charts, e.g., “Table 3b: Pre-CLARP intervention balance check of outcome variables is a balance check of outcome variables pre-CLARP.”
- I think tables should appear numerically; as it is table 3b comes before table 3a
- Across the board, when referring to quantitative results, please indicate the p-values from table 3a, or other statistics from the other tables such as the means; at least the significant ones should be shown in the text, not just in the table referrals
- Lines 537-539: what is meant by “DEQ” here? I am presuming these are codes for the results.
- Lines 536-543: The text here is repetitive/duplicated; it is introducing the results twice
- In section 3.6.2b authors highlight an economic benefit that the community sees from the FGM/C. Similar to polygamy some communities find value which has been quantitatively demonstrated in a major study from across the border in Tanzania that presents “**counterevidence** that polygyny is often positively associated with food security and child health within communities” [Lawson DW, James S, Ngadaya E, Ngowi B, Mfinanga SG, Borgerhoff Mulder M. No evidence that polygynous marriage is a harmful cultural practice in northern Tanzania. *Proc Natl Acad Sci USA*. 2015 Nov 10;112(45):13827-32]

Discussion:

- Lines 657, 666 and elsewhere speak of “CLARP models”. So far in the previous sections as a reader I assumed CLARP was only referring to the AMREF model implemented in Kajiado. As such, saying “models” warrants a call for authors to mention the countries or other sites where the model (were these similar) has been implemented?
- Other than CLARP and YIDA, are there other models that have been used elsewhere with similar effects even if in a single outcome like reducing prevalence of FGC/M? more examples and comparisons lend credence to these interventions; and could catalyze a need for a systematic review (even a Cochrane). Talking about a Cochrane, one I could find could be a good reference for assessing pregnancy outcomes among FGM/C women [Balogun OO, Hirayama F, Wariki WM, Koyanagi A, Mori R. Interventions for improving outcomes for pregnant women who have experienced genital cutting. *Cochrane Database Syst Rev.* 2013 Feb 28;2013(2):CD009872.]

Limitations:

- The DiD approach used for the quantitative analysis and the qualitative analysis may not rule out effect of other interventions or changes. Overall, FGM/C has dropped across the country, even in the control group of counties. Thus, attributing the intervention alone is still a challenging conclusion to defend. For instance, I am aware that Ms Nice Nailantei, a 27-year-old anti-FGM activist from Oloitokitok, Kajiado South, made it to the TIME magazine’s 2018 Top 100 Most Influential list [<https://time.com/5253582/nice-nailantei-lengete-time-100-gala/>]. No doubt her work (separate and independent of AMREF’s CLARP) would also have contributed to accelerate the decline in FGM/C and the reported outcomes. A point of successful model women is made in lines 617-623. Ms. Nailantei is one model that can have significant effect on girls in the community under study.
- Reporting of the practice and attitudes through interviews will also have limitations. Authors note that the practice is reported to be taking place in secrecy (subsection 3.6d). If the KDHS data and the reinforcing reports of success reported through CLARP are believable then the practice is still declining, which should raise less concern of the widespread secret circumcisions
- The harms of FGM/C should be demonstrated quantitatively (section 3.6.2c). While not an area of my work I have yet to see a study to quantitatively show the claimed medical harms – e.g., increased STIs and complications in child-birth. See, e.g., [The Public Policy Advisory Network on Female Genital Surgeries in Africa. **Seven Things to Know about Female Genital Surgeries in Africa**. *Hastings Cent Rep*. 2012 Nov-Dec;42(6):19-27.] I have thought that if researchers can show the community these medical harms it will be additional evidence to the psychosocial concerns. The community has been practicing FGC for a long time and change in a cultural practice can take a long time. Among the Kikuyu’s in central Kenya generalized change in the practice took a relatively very short time from the time first President Jommo Kenyatta wrote Facing Mount Kenya in the 1930s to the next few decades, the practice was almost gone at the time of independence [Frederiksen BF. Jomo Kenyatta, Marie Bonaparte and Bronislaw Malinowski on clitoridectomy and female sexuality. *Hist Workshop J*. 2008 Spring;65:23-48.] In the case of the Kikuyu, education was the primary approach to ending the practice

Conclusion:

- In view of the above concern on limitations, and those you have indicated, I suggest conditioning the term “decisive” used in line 758. This is also not quantitatively validated. It is better to use p-value validation, terms like “significant”.
- Broader utility of the CLARP and the results beyond Kajiado should be stated. At the very least authors should be able to venture a utility in other Maasai communities in Kenya and Tanzania. For example, can it work in Narok county in Kenya? Outside the Maasai communities (like the YIDA model) elsewhere in Kenya and the African sub-region, do the authors think it has utility (scalability and replicability)? AMREF works in many parts of Africa; why not implement it more widely? At the least authors should say this. And upon possible publication of this paper the authors should continue their work and study this further for outstanding questions such as these and others.

References:

- For the Background section few peer-reviewed studies on the FGM/C practice are cited. Are there no such studies, observational studies, or perhaps even academic dissertations?

Figures:

- Have the authors obtained permissions for maps in Figure 1a and 2b?
- It would be ideal to add legends to figures so these are self-explanatory and reader does not have to go to the text to look for what these depict

Grammar:

- Minor errors need to be checked. For example, a term is missing in line 87-88 “Second, complications during pregnancy and childbirth and continue…” ; line 459 and table 3c should “sign” not be “size” for ‘effect size’? In line 571 “practise” should be “practice”

Funding declaration:

- The statement that “The funders reviewed the manuscript and provided approval for publication” could be potentially problematic. Why would the funders play such a role? Ordinarily funders do not play any role in authors decision to write the manuscript or submitting it for peer-review.
