# Supplementary material for: The impact of community led alternative rite of passage on eradication of female genital mutilation/cutting in Kajiado County, Kenya: A quasi-experimental study
Source: PLoS One. 2021 Apr 28;16(4):e0249662. doi: 10.1371/journal.pone.0249662 (PMC8081212; doi:10.1371/journal.pone.0249662)
Supplement: Attachment — Submitted filename: Response to Reviewers.docx [file pone.0249662.s003.docx]

| **Comment** | **Response** |
| --- | --- |
| Please ensure that your manuscript meets PLOS ONE's style requirements, including those for file naming. The PLOS ONE style templates can be found at <https://journals.plos.org/plosone/s/file?id=wjVg/PLOSOne_formatting_sample_main_body.pdf> and  <https://journals.plos.org/plosone/s/file?id=ba62/PLOSOne_formatting_sample_title_authors_affiliations.pdf> | The comment is well appreciated. The manuscript sections have been revised to follow the journal format |
| Please include additional information regarding the survey or questionnaire used in the study and ensure that you have provided sufficient details that others could replicate the analyses. For instance, if you developed a questionnaire as part of this study and it is not under a copyright more restrictive than CC-BY, please include a copy, in both the original language and English, as Supporting Information. | The study utilized various tools to collect qualitative data. We have attached the following study tools used:   1. IDI guide for ARP beneficiaries 2. IDI guide for non-ARP Beneficiaries 3. KII guide for Chief 4. KII guide for member of County Assembly 5. KII guide for Child protection officer 6. KII guide for Headteacher 7. KII guide for CBO Official 8. KII guide for Cultural leaders 9. KII guide for Religious leaders 10. KII guide for TBA- Cutters 11. FGD guides for adolescent girls and Young women 12. FGDs guides for *Morans* and young women 13. FGDs guides for parents |
| Your ethics statement should only appear in the Methods section of your manuscript. If your ethics statement is written in any section besides the Methods, please move it to the Methods section and delete it from any other section. Please ensure that your ethics statement is included in your manuscript, as the ethics statement entered into the online submission form will not be published alongside your manuscript. | As recommended, the ethics statement titled “Ethics approval and consent to participate” has been moved to section 2.4 under Materials and methods |
| We note that [Figure(s) 1a, 1c, 2a and 2b] in your submission contain map images which may be copyrighted. All PLOS content is published under the Creative Commons Attribution License (CC BY 4.0), which means that the manuscript, images, and Supporting Information files will be freely available online, and any third party is permitted to access, download, copy, distribute, and use these materials in any way, even commercially, with proper attribution. For these reasons, we cannot publish previously copyrighted maps or satellite images created using proprietary data, such as Google software (Google Maps, Street View, and Earth). For more information, see our copyright guidelines: <http://journals.plos.org/plosone/s/licenses-and-copyright>. | We have removed all images which might be copyrighted and left only those constructed by the authors |
| We require you to either (1) present written permission from the copyright holder to publish these figures specifically under the CC BY 4.0 license, or (2) remove the figures from your submission: | We have removed all images which might be copyrighted and left only those constructed by the authors |
| You may seek permission from the original copyright holder of Figure(s) [a, 1c, 2a and 2b] to publish the content specifically under the CC BY 4.0 license. | We have removed all images which might be copyrighted and left only those constructed by the authors |
| Background: Overall: Much attention is paid in this section to descriptive statistics surrounding FGM/C in Kenya. Although surely relevant, I’d rather see a steady build up to the problem statement. What is CLARP (rationale, history, and so on and so forth)? Why has it taken so long for decent effect studies to be conducted? What are the challenges involved? What is at stake, also for the NGO itself? | The section on CLARP and relevant details have been included |
| Line 28-29: It is recorded that Amref Health Africa started implementing ARPs – not CLARPs – in 2009. However, credits for the idea and design of this intervention should likely also be given to MYWO that already implemented an ARP in 1996, well before Amref Health Africa started implementing its own version. Furthermore, the authors here do not sufficiently explain and expand upon the intervention, the underlying rationale for the intervention, the applicability of the intervention in communities in which ARP does not primarily function as a rite of passage into womanhood and the added value of CL in the acronym CLARP. Isn’t CL already implied in ARP? In the end, it is not about claiming new acronyms (see also: Billig, 2013), but about actual change practices in the field. | The comment is well appreciated and details included in section 1.2 of the introduction |
| Later in the paper (Line 398: 2.5 An overview of the CLARP model) the authors do expand upon CLARP in more detailed fashion. I would suggest integrating this section in the introduction, as to help the reader to better understand the underlying rationale for the CLARP approach, and this research project into its effectiveness | The comment is well appreciated, and details included in section 1.2 of the introduction |
| . Line 59-90: “… FGM/C is still practised as a social norm and thus remains persistent and ubiquitous across countries.” This is kind of a casual explanation – by the use of the word ‘thus’ – of FGM/C’s persistency, but a debatable one too (e.g., Efferson et al., 2015; Hughes, 2018). | The sentence has been revised to read “… FGM/C is still practiced as a social norm and remains persistent and ubiquitous in some communities across countries” |
| Line 77 \| 140: “… paints a gloomy picture …”. In presenting numbers and figures I’d prefer more neutral language. | The sentence has been deleted |
| Line 93-94: “Finally, going through the FGM/C ordeal and the resultant psychological effects can have devastating effects on girls’ and women’s mental health.” This statement is certainly not disputed, at least not by me. However, there is a flip-side to it. Exaggerating or over-emphasizing negative (mental) health effects, or not placing these in the context of the mental health effects suffered from not-being circumcised in a community that traditionally practices it, can have devastating effects on the credibility of health workers working in these communities (e.g., Shweder, 2000). Bottom line, what do the authors precisely mean when they employ the word ‘devastating’? Words matter, and can become self-fulfilling prophecies, i.e., leading to stigmatization of circumcised girls, circumcised girls giving up on exploring for sexual pleasure/orgasms, etcetera. These dynamics may not be papered over, because these help to understand FGM/C’s persistency (see also previous point with regards to oversimplifying these dynamics under a, casually and causally used, yet purely descriptive social norm). | The statement has been deleted |
| Line 183: “ … primarily … “ (?). Would the intervention and underlying rationale be valid in communities in which FGM/C is practiced for different reasons, or with a different functional emphasis in mind (e.g., Gruenbaum, 2001)? And, who says FGM/C functions primarily as a rite of passage, even in the communities Amref Health Africa works with. Recently, some doubts have been raised and nuances made, particularly with regards to current practices in Maasai communities (e.g., Van Bavel et al., 2017; Van Bavel, 2020).. | The sentence has been deleted |
| Line 187-189: Again, the CLARP acronym might be relatively new – introduced well after 2009 even, but the intervention itself is not. The claim here ignores the contribution of other organizations that have been experimenting with these types of interventions well before 2009, and outside of Kajiado County | Recognition of the introduction of ARP by *Maendeleo ya Wanawake* in 1996 has been included in section 1.2 of introduction |
| Line 199: “… stands out as one of the essential community-led initiatives …”. Who says so? Since many of the authors appear to be affiliated with Amref Health Africa, a more tentative tone is likely warranted. This in order to demonstrate to readers that the authors clearly distinguish between how the intervention is marketed towards donors and other stakeholders, and that what is actually known to work – which is still very little (Droy et al., 2018). Expanding upon this more thoroughly would help justifying this research project and the chosen methods. Fairly recently World Vision – another NGO that implements ARPs in the same regions in which Amref Health Africa is active – has been criticized for the way they’ve marketed their ARP, its design, its implementation and lack of access for independent researchers to study impact (Hughes, 2018). | This has been revised to include this among other ongoing interventions to reduce FGM C in Kajiado |
| Line 204-206: “However, despite the declining trends in FGM/C prevalence, the extent to which the CLARP model had a role to play in such declines in Kenya, and particularly in Kajiado county remains unclear.” That is correct. So I guess readers likely expect a detailed elaboration in this introductory paragraph – after outlining what kind of practices underlie the acronym CLARP – on why this is so, since the intervention is implemented for well over a decade now. Eminent scholars on FGM/C, such as Bettina Shell-Duncan, may surely be referred to in support of the idea, but there are also those scholars with a more particular focus on ARP, or CLARP if you will, that have been critiquing the lack of evidence regarding program effectiveness that may not be ignored (e.g., Droy et al., 2018; Hughes, 2018). The authors should do all they do can to avoid being accused of cherry picking literature that favors CLARP. | A description of CLARP has been included in the introduction |
| Line 215-218: A firm theoretical underpinning of what is meant with experiences, stories or narratives, attitudes, perceptions and practices, and how they all relate or differ is lacking. So it is unclear of what the authors are looking for exactly. | The study sought to explore experiences and perceptions of ARP beneficiaries and non-beneficiaries with regards to how ARP has influenced FGM/C practice, Early marriages. Teenage pregnancies and school attendance |
| Overall: Especially with regards to the qualitative part of the study, more information is warranted of what the researchers have done to reduce their own (confirmation) bias, and to account for social desirability in their respondents’ reactions. Most of the authors appear to be affiliated with Amref Health Africa and respondents, in some cases, may be dependent on the health care services this NGO provides for them. It is not unlikely respondents have stake in appeasing their interviewers, or in the outcomes of this project. How did the researchers account for these tendencies? What measure did they take? | The researchers engaged 3 independent consultants to conduct the impact evaluation. They were involved in proposal development, data collection and analysis. There was minimal contact of the Amref Health Africa staff with the study participants during the interviews hence minimizing the respondents bias owing to benefits of the program. This has been included on line 403-408 |
| Line 253: “… non-existent … ”. Taking into account the proliferation of similar interventions being implemented by a number of NGOs and lack of coordination among them, how can we be sure? Bottom line, on which sources or data is this claim based. Who has the data? The Kenyan Anti-FGM Board? | A desk review of reports from the counties and existing NGOs indicated that such CLARP intervention or a similar one, as late as 2015, was non-existent in these counties. This has been updated on 263-266 |
| Line 344: “… purposive … “. Please specify sampling strategy. Purposive sampling is an umbrella term for a range of strategies used in qualitative research (e.g., Palinkas et al., 2015) | This has been elaborated on line 358-366 |
| Line 373: Integrate in sampling paragraph. | This has been integrated in section 2.2.2: Data collection |
| Line 398: Move to introduction. | Done |
| I’d recommend to give more body to the discussion paragraph, for example by being more self-reflective about the research process. | We have added a paragraph on this, lines 903-914 |
| In writing up the limitations paragraph the paper written by Ian Askew (2005) might come in handy. I think this research knows quite a number of limitations that need to be accounted for, especially in light of the final conclusion drawn, that is in definite favor of the CLARP intervention. | More detail is added in section 2.5. Study limitations |
| Line 758-759: “The study revealed that CLARP played a decisive role in attenuating FGM/C, CEFM and TP in Kajiado County.” Especially the word ‘decisive’ makes this a very firm statement. As yet, based on the information the authors provide, I am not convinced this firm statement is warranted. However, I would be happy to be proven wrong | Statement has been revised |
| This manuscript is a quasi-experimental study of community-based interventions on female genital mutilation/cutting (FGM/C) in one region of Kenya among the Maasai who have traditionally conducted the practice universally. The topic of the study is of importance to public health and is also a stated goal of the 2030 Sustainable Development Agenda (goal 5.3). As important as the topic is, it seems there is limited empirical evidence of the effect of FGM/C, or that the evidence is not clear-cut to warrant use of terms like “devastating”. For this, I would advise researchers to consider arguments made in this report [The Public Policy Advisory Network on Female Genital Surgeries in Africa. Seven Things to Know about Female Genital Surgeries in Africa. Hastings Cent Rep. 2012 Nov-Dec;42(6):19-27.] | The paper suggested below is a good one from the abstract. We could however not access the full read as it is restricted. We have reviewed the language in the paper to do away with strong terms such as “devastating” |
| Abstract  Authors should add more details on the Methods statement here. For instance, what type of data is used, where is the data from (e.g., KDHS), what is the sample size, what is the analytical method used (e.g., the DiD)? All that is now missing. The single statement there now on the design of the study is highly uninformative. In general, it is puzzling that the authors seem not to have thought it necessary to include all these other details  In the Conclusion sub-section more could be stated. For instance, is the CLARP scalable, is it replicable, what is the policy implication? | We have updated the abstract to capture more information as suggested while also balancing not to exceed the word count |
| Lines 61-63: can authors add references to this statement | References have been included |
| Lines 76-84: the UNICEF source (and for Figure 1a) that is mentioned here should be cited | References have been included |
| Lines 85-86: cite the UNFPA document here first | The section has been deleted |
| Lines 87-89: the statement says that FGM/C complicates pregnancy and childbirth and “continue to be the leading cause of death among 15- to 19-year-old girls in most parts of the developing world.” There is some contradiction with the statement “in most parts of the developing world” because a previous line (77-79) state the practice is “concentrated across 29 countries”. This is not “most of the developing world”. | The section has been deleted |
| Lines 116-120: does not the KDHS capture types of FGM/C? The quality of references used do not seem strong; later in lines 134-138 data on the different types are noted from the KDHS. | KDHS 2014 Reference is included |
| I think section 1.3 (objectives) and 1.4 (evaluation questions) are repetitive and one can be dropped | Evaluation questions deleted. |
| Mention here in this section that ending FGM/C is also part of the Sustainable Development Agenda to 2030 where Goal 5.3 specifically mentions the practice [<https://sdgs.un.org/goals/goal5>] . | This has been added |
| - The variables “religion” and “ethnicity” are included in the model. Table 2a with KDHS data does not show these, but it is presumed data for these come from the same KDHS; perhaps include these in this Table as well | Added |
| - Can the authors include sample questionnaires as supplemental files? Since the direct questions are not shown in the results – responses are included – the description of what was contained in these questionnaires under “2.3.2-Data collection tools” is not sufficiently informative | included |
| How have the authors dealt with data from KDHS in years prior to implementation of the County system in 2010? The analysis of quantitative data between treatment county (Kajiado) and control group of counties for all indicators extracted from KDHS would have been collected using regional samples that are different from the county frame. Are the authors using the district frame or other county equivalence? Table 3a seem to show regions by District/sub-county | We have used District/Sub-county as the variable prior to 2010 and after. |
| In addition to the KDHS, data from the schools – on educational outcomes/schooling years, completion and retention – in the intervention county (Kajiado) could also be collected and analyzed quantitatively as the records could be available. Perhaps this is still an open research question | We tried this option but encountered numerous gaps and inconsistencies in the data from schools and could not continue |
| Is it known what the number of beneficiaries of CLARP was? Lines 341-342 states AMREF has a database but the sample was derived “within a school from randomly drawn list of schools” (lines 333-334). The sample size is not shown or stated | Not sure whether we got the comment right, but we have included a sample of the beneficiaries drawn for IDIs in line 351-357 |
| Please check duplicated text in stating your charts, e.g., “Table 3b: Pre-CLARP intervention balance check of outcome variables is a balance check of outcome variables pre-CLARP.” | Duplicate text deleted |
| I think tables should appear numerically; as it is table 3b comes before table 3a | Done |
| Across the board, when referring to quantitative results, please indicate the p-values from table 3a, or other statistics from the other tables such as the means; at least the significant ones should be shown in the text, not just in the table referrals | We have captured the p-values to support each of the qualitative findings |
| Lines 537-539: what is meant by “DEQ” here? I am presuming these are codes for the results. | The code has been removed |
| Lines 536-543: The text here is repetitive/duplicated; it is introducing the results twice | The text has ben deleted. |
| In section 3.6.2b authors highlight an economic benefit that the community sees from the FGM/C. Similar to polygamy some communities find value which has been quantitatively demonstrated in a major study from across the border in Tanzania that presents “**counterevidence** that polygyny is often positively associated with food security and child health within communities” [Lawson DW, James S, Ngadaya E, Ngowi B, Mfinanga SG, Borgerhoff Mulder M. No evidence that polygynous marriage is a harmful cultural practice in northern Tanzania. *Proc Natl Acad Sci USA*. 2015 Nov 10;112(45):13827-32] | The comment on economic value of polygamy is noted. This section is highlighting what the participants think is the economic value of circumcision |
| Lines 657, 666 and elsewhere speak of “CLARP models”. So far in the previous sections as a reader I assumed CLARP was only referring to the AMREF model implemented in Kajiado. As such, saying “models” warrants a call for authors to mention the countries or other sites where the model (were these similar) has been implemented? | These have been corrected |
| Other than CLARP and YIDA, are there other models that have been used elsewhere with similar effects even if in a single outcome like reducing prevalence of FGC/M? more examples and comparisons lend credence to these interventions; and could catalyze a need for a systematic review (even a Cochrane). Talking about a Cochrane, one I could find could be a good reference for assessing pregnancy outcomes among FGM/C women [Balogun OO, Hirayama F, Wariki WM, Koyanagi A, Mori R. Interventions for improving outcomes for pregnant women who have experienced genital cutting. *Cochrane Database Syst Rev.* 2013 Feb 28;2013(2):CD009872.] | We did not find any other interventions apart from CLARP and YIDA. We also reviewed the paper on “Interventions for improving outcomes for pregnant women who have experienced genital cutting” and the paper did not document any intervention for improving outcomes for those who have undergone FGM/C |
| The DiD approach used for the quantitative analysis and the qualitative analysis may not rule out effect of other interventions or changes. Overall, FGM/C has dropped across the country, even in the control group of counties. Thus, attributing the intervention alone is still a challenging conclusion to defend. For instance, I am aware that Ms Nice Nailantei, a 27-year-old anti-FGM activist from Oloitokitok, Kajiado South, made it to the TIME magazine’s 2018 Top 100 Most Influential list [<https://time.com/5253582/nice-nailantei-lengete-time-100-gala/>]. No doubt her work (separate and independent of AMREF’s CLARP) would also have contributed to accelerate the decline in FGM/C and the reported outcomes. A point of successful model women is made in lines 617-623. Ms. Nailantei is one model that can have significant effect on girls in the community under study. | We have acknowledged DiD approach as not the best method to fully tease out the impacts of intervention. It is however the most appropriate design given that experimentation was not integrated in the design right from the start of the programme.  The work Ms Nailantei is part of the CLARP programme and not a separate model that can be sighted on its own. |
| Reporting of the practice and attitudes through interviews will also have limitations. Authors note that the practice is reported to be taking place in secrecy (subsection 3.6d). If the KDHS data and the reinforcing reports of success reported through CLARP are believable then the practice is still declining, which should raise less concern of the widespread secret circumcisions | The aspect of reporting on practices using qualitative interviews is noted and included in study limitations. See line 461-462 |
| The harms of FGM/C should be demonstrated quantitatively (section 3.6.2c). While not an area of my work I have yet to see a study to quantitatively show the claimed medical harms – e.g., increased STIs and complications in child-birth. See, e.g., [The Public Policy Advisory Network on Female Genital Surgeries in Africa. **Seven Things to Know about Female Genital Surgeries in Africa**. *Hastings Cent Rep*. 2012 Nov-Dec;42(6):19-27.] I have thought that if researchers can show the community these medical harms it will be additional evidence to the psychosocial concerns. The community has been practicing FGC for a long time and change in a cultural practice can take a long time. Among the Kikuyu’s in central Kenya generalized change in the practice took a relatively very short time from the time first President Jomo Kenyatta wrote Facing Mount Kenya in the 1930s to the next few decades, the practice was almost gone at the time of independence [Frederiksen BF. Jomo Kenyatta, Marie Bonaparte and Bronislaw Malinowski on clitoridectomy and female sexuality. *Hist Workshop J*. 2008 Spring;65:23-48.] In the case of the Kikuyu, education was the primary approach to ending the practice | This is out of scope for the study. However, it is a very important component to be considered for future studies |
| In view of the above concern on limitations, and those you have indicated, I suggest conditioning the term “decisive” used in line 758. This is also not quantitatively validated. It is better to use p-value validation, terms like “significant”. | Done |
| Broader utility of the CLARP and the results beyond Kajiado should be stated. At the very least authors should be able to venture a utility in other Maasai communities in Kenya and Tanzania. For example, can it work in Narok county in Kenya? Outside the Maasai communities (like the YIDA model) elsewhere in Kenya and the African sub-region, do the authors think it has utility (scalability and replicability)? AMREF works in many parts of Africa; why not implement it more widely? At the least authors should say this. And upon possible publication of this paper the authors should continue their work and study this further for outstanding questions such as these and others. | CLARP is replicable as it is currently it is being implemented in Tanzania. We recommend scaling it up for adoption by stakeholders implementing in other counties that practice FGM/C in Kenya and across other sub Saharan Africa countries as a rite of passage. This has been included in the conclusion |
| References:  For the Background section few peer-reviewed studies on the FGM/C practice are cited. Are there no such studies, observational studies, or perhaps even academic dissertations? | We have included more peer reviewed studies that we found relevant in the context of the paper. |
| Figures:   - Have the authors obtained permissions for maps in Figure 1a and 2b?   It would be ideal to add legends to figures so these are self-explanatory and reader does not have to go to the text to look for what these depict | All images that might need permission to publish have been deleted  The figures have been properly referenced and legends added |
| Minor errors need to be checked. For example, a term is missing in line 87-88 “Second, complications during pregnancy and childbirth and continue…” ; line 459 and table 3c should “sign” not be “size” for ‘effect size’? In line 571 “practise” should be “practice” | We have run through the content and corrected such errors |
| Funding declaration:  The statement that “The funders reviewed the manuscript and provided approval for publication” could be potentially problematic. Why would the funders play such a role? Ordinarily funders do not play any role in authors decision to write the manuscript or submitting it for peer-review. | The statement has been deleted |
