# Supplementary material for: The impact of community led alternative rite of passage on eradication of female genital mutilation/cutting in Kajiado County, Kenya: A quasi-experimental study
Source: PLoS One. 2021 Apr 28;16(4):e0249662. doi: 10.1371/journal.pone.0249662 (PMC8081212; doi:10.1371/journal.pone.0249662)
Supplement: Attachment — Submitted filename: Response to Reviewers.docx [file pone.0249662.s004.docx]

| **Comment** | **Response** |
| --- | --- |
| 81 ‘with traditional practitioners being the main perpetrators of the practice.’ Maybe specify. In contrast to trained medical professionals? What is implied here? | We have clarified this by adding to the statement below:  79 “with traditional practitioners such as cultural elders and female circumcisers/Traditional Birth Attendants (TBAs) being the main perpetrators of the practice” |
| 185 An interested layman might not know what ‘Morans’ are? | We have clarified this by adding to the statement below:  184 “ *Morans* ( young *Maasai* men who live in isolation in bushes, learning tribal customs and developing strengths, courage and endurance —traits for which Maasai warriors are known for throughout the world)” |
| 189 Same for the acronym TBA. Please clarify | 80 TBA has been written in full as Traditional Birth Attendants at the first point of mention in line 82 |
| 196 Same for the acronym CSO. Please clarify by using APA or journal recommended standards for employing acronyms | 197 CSO has been written in full as Civil Society Organization at the first point of mention in line 197.  We have also run through the whole manuscript to ensure that all acronyms are written in full at first mention |
| 359 What type of purposive sampling? Related to an earlier review, I see it is addressed, but is this a particular tried sampling strategy (e.g., homogeneous sampling, typical case sampling, extreme/deviant case sampling, maximum variation sampling, critical case sampling, total population sampling, expert sampling)? | 360 This has been specified as homogeneous purposive sampling |
| 365 ‘Barazaas’? These notions are obvious to the authors, but likely not to most readers. | 368 This has been clarified as public meetings |
| 373 ‘… male and female parents’. I don’t know whether this is a pleonasm, but close to it. | Not necessarily a pleonasm. What we mean here is that FGDs will be organized with male and female parents separately. Same to young men and women and other sub-groups. |
| 375 Please clarify what a ‘Moran’ is immediately when first introducing the term (line 185). This will enhance readability of the paper. | Done |
| 461 ‘The aspect of reporting on practices using qualitative interviews is limiting as the practice is done in secrecy.’ Sure, but this is also the case for quantitative surveys methods. What steps have been taken to deal with secrecy, possible risks for participants and the double-bind of gaining rapport and confidentiality? | 460-464 We have included the measures taken to gain rapport and confidentiality of the respondents to freely share information during qualitative interviews.  “This was however mitigated through assuring and re-assuring study participants during consenting that any information shared will be kept confidential to the fullest extent of the law and that the study team will not discuss any information about them or what they shared unless with written permission”  The reason why quantitative surveys is not included as a limitation is that we used already collected KDHS data and therefore we did not collect any quantitative data to respond to the study objectives |
| 465. ‘… given that experimental design was not integrated in the programme right from the start of the implementation.’ Why not? | 468-470 Added a statement to qualify why experimental design was not integrated in the programme right from the start of the implementation  “… the implementation as it would have been impractical to do so given that we did not have control over implementation of similar activities by other organizations” |
| 508-514 Not sure whether this quotation is exactly on point. CLARP or ARP is not mentioned, and generally speaking, people in Eastern African communities understandably like Amref Health Africa to be active and remain in their region for their work on WASH and public health more broadly. ARP for some of the community members is a great lever to address these broader issues as well. | We captured the quote as it was from the Moran with the understanding that this was quoted in the context of CLARP  We hope this clarifies |
| §3.5 Experiences and stories of CLARP versus non- CLARP beneficiaries The title of this paragraph overpromises and underdelivers. I can imagine a lot more rigorous work underlies this paragraph, but it does not show here. It also stands in contrast to §3.6. Perhaps the authors want to stuff too much into one single paper. They might want to consider writing another paper to do justice to the underlying stories collected and experiences shared. Otherwise, I’d recommend a rewrite of this paragraph | As recommended we have dropped the section on Experiences and stories of CLARP versus non- CLARP beneficiaries and the whole objective related to this for consideration in a different paper. |
| §3.6 Perceptions, attitudes and practices of community stakeholders about FGM/C practices My main point of critique here is that key notions (e.g., stigma reduction, culture change) are not defined and mirrored against the existing body of literature. | 824-831  We have included a paragraph, including some literature to mirror some of the barriers identified in the study |
| 3.6.2 Opportunities to improve/adapt Please mirror these findings against existing academic literature on FGM prevention. | We feel that the opportunities to improve/adapt as highlighted in the paper are mirrored in the paragraph added in lines 931-945, especially on misperceptions about uncircumcised girls and economic empowerment. We have in different sections of this paper highlighted on law enforcement and political will done through designing strategies, plans, policies and passing laws against the practice. |
| 817 The acronyms ARP and CLARP are used interchangeably here. This is confusing to the reader. Are ARP and CLARP different interventions? Please clarify. | In the discussion section, we have used ARP when referring to evidence in existing body of literature, while used CLARP when talking about the intervention that we are sharing its evidence. We have read through and there is no interchangeable use of the acronyms |
| 877 I would repeat here what was rightfully mentioned earlier … in those communities wherein FGM/C is practiced as a rite of passage into womanhood, or something along those lines. | We did not understand this comment and therefore did not do anything to it |
| 891 ‘… act as a safe bet’ Please avoid employing metaphorical language. | 873 We have changed this statement to “…act as a replacement for …” |
| 894 ‘alarming rates’ Let the data speak for itself. | 876 “alarming” is relative and therefore to us CEFM rates above 20% as shown in the quoted references is alarming. We have therefore not changed anything here |
| 900-901 ‘Amref Health Africa’s noble work’ Since this is an academic paper please do not turn it into an Amref pamphlet (Amref is mentioned > 20 times), or make it appear as such. That would discredit the great work underlying this paper. That is not to say, of course, that Amref’s work is not noble. However, noble is irrelevant. | 881-884 We have revised the statement to read  “The contribution of CLARP interventions in the fight against FGM/C practices and their related manifestations suggest more resources and collaborative efforts from other stakeholders are needed to scale-up the CLARP interventions to eradicate FGM/C” |
| Minor points  Use of acronyms, typos, capital letters, and so on and so forth | We have run through the paper to correct these mistakes |
